# Vaccination of Mice with Virulence-Associated Protein G (VapG) Antigen Confers Partial Protection against *Rhodococcus equi* Infection through Induced Humoral Immunity

**DOI:** 10.3389/fmicb.2017.00857

**Published:** 2017-05-11

**Authors:** Marcel M. Trevisani, Ebert S. Hanna, Aline F. Oliveira, Silvia A. Cardoso, Maria C. Roque-Barreira, Sandro G. Soares

**Affiliations:** Departamento de Biologia Celular e Molecular e Bioagentes Patogênicos, Faculdade de Medicina de Ribeirão Preto, Universidade de São PauloSão Paulo, Brazil

**Keywords:** *Rhodococcus equi*, vectored vaccine, VapG, APTX, attenuated *Salmonella*

## Abstract

*Rhodococcus equi* is a facultative intracellular bacterium causing severe pyogranulomatous pneumonia, ulcerative enterocolitis, and mesenteric lymphadenopathy in foals aged less than 6 months. Less frequently, this pathogen affects various other species, such as pigs, cattle, cats, and even humans. Although rhodococcosis is treated with a combination of antimicrobial agents, resistance is developed in some cases, and thus, antimicrobial susceptibility must be monitored and managed. Considering these limitations of the current therapy and unavailability of a vaccine to prevent the disease, research is particularly focused on the development of an effective vaccine against rhodococcosis. Most vaccines undergoing development utilize the virulence-associated protein (Vap) A antigen, which was identified previously as a key virulence factor of *R. equi*. Nevertheless, other proteins, such as VapG, present in most virulent *R. equi* strains, are also encoded by vap genes located on the *R. equi* bacterial virulence plasmid. In the present study, we evaluated the effect of VapG immunization on the survival of *R. equi*-challenged mice. We used attenuated *Salmonella* as a carrier for VapG (*Salmonella*-*vapG*+), a procedure previously adopted to develop a VapA-based vaccine. We observed that vaccination with *Salmonella*-*vapG*+ induced both an increased IFN-γ, IL-12, and TNF-α production, and a decreased bacterial burden in organs of the *R. equi*-challenged mice. Nevertheless, *Salmonella*-*vapG*+ vaccination protected only 50% of the mice challenged with a lethal dose of *R. equi*. Interestingly, we observed an increased frequency of B cells in the spleen of *Salmonella*-*vapG*+-vaccinated mice and showed that *Salmonella*-*vapG*+-vaccinated *R. equi*-challenged B-cell-knockout mice did not reduce the bacterial burden. Given these results, we discussed the potential role of the humoral immune response induced by *Salmonella*-*vapG*+ vaccination in conferring protection against *R. equi* infection, as well as the employment of VapG antigen for obtaining hyperimmune plasma to prevent rhodoccocosis in young foals.

## Introduction

*Rhodococcus equi* (*Rhodococcus hoagii/Prescottella equi*) is an important equine pathogen leading to a high mortality rate in newborn foals, thereby exerting a major financial impact on the equine industry (Venner et al., 2012). This gram-positive facultative intracellular bacterium causes severe pyogranulomatous pneumonia, as well as other less frequent clinical conditions, such as ulcerative enterocolitis and mesenteric lymphadenopathy (Giguere and Prescott, 1997). Mortality rates for *R. equi*-infected untreated foals range from 70 to 80%, while the treatment of infected foals decreases this rate, which persists at 30% (Giguere et al., 2004; Coulson et al., 2010). Furthermore, *R. equi* has been reported to frequently infect pigs, and to occasionally infect cattle, cats, and dogs. Although healthy humans are rarely infected with *R. equi*, immunocompromised individuals are susceptible to the infection (Yamshchikov et al., 2010). Currently, vaccines against rhodococcosis are unavailable commercially, in spite of the significant investment of international resources to develop an effective *R. equi* vaccine (Giles et al., 2015).

*Rhodococcus equi* harbors an 80–90-kb plasmid encoding virulence-associated proteins (Vaps) that enable the bacterium to survive, persist, and replicate within the host macrophages (Zink et al., 1987). The plasmid comprises of six full-length vap genes (*vapA*, -*C*, -*D*, -*E*, -*G*, and -*H*) and three vap pseudogenes (*vapF*, -*I*, and -*X*), whose coding sequences are either truncated or mutated (Giguere et al., 2004). All virulent *R. equi* strains isolated from infected foals were reported positive for VapA, a bacterial surface lipoprotein required for intracellular growth in the macrophages. Promisingly, deletion of *vapA* has been shown to attenuate the virulence of *R. equi* strains (Jain et al., 2003). Nevertheless, expression of VapA alone is insufficient to facilitate virulence, as demonstrated by Giguere et al. (1999), who studied that the introduction of exogenous wild-type *vapA* into a plasmid-cured *R. equi* strain was not sufficient to restore bacterial virulence, a fact that was demonstrated either in a murine model of *R. equi* infection or in challenged foals. Thus, additional factors are required to facilitate the ability of *R. equi* to colonize tissues and provoke clinical symptoms in foals, as indicated in several studies: (i) Ren and Prescott (2003) showed that all vap genes are expressed in *R. equi* isolated from macrophages of infected equines; (ii) Monego et al. (2009) showed that VapA, VapG, and VapD are present in all the analyzed isolates from clinical samples; (iii) Benoit et al. (2002) demonstrated that the expression of *vapA* and *vapG* can be induced by H_2_O_2_ treatment, suggesting that these genes exert a protective effect against macrophage-related stresses; (iv) Jacks et al. (2007) observed an augmented expression of *vapA, vapD*, and *vapG* in bacteria isolated from the lung tissue of infected foals, suggesting that these genes are implicated in *R. equi* pathogenesis. Together, these results indicate the importance of considering all vap genes as candidates for vaccine components.

Previous studies have demonstrated that the VapA antigen carried by attenuated *Salmonella enterica* Typhimurium (*Salmonella-vapA*+) induces a protective immune response in *R. equi*-challenged mice (Oliveira et al., 2007, 2010). In the current study, we assessed the ability of the vaccine with VapG-antigen carried by attenuated *S. enterica* Typhimurium (*Salmonella*-*vapG*+) to protect mice against *R. equi* infection.

## Materials and Methods

### Ethics Statement

The study was performed according to the norms established by the National Council for the Control of Animal Experimentation (CONCEA). The protocol of the study was approved by the Ethics Committee on Animal Research of the University of São Paulo (USP) (protocol 107/2011).

### Mice, Bacterial Strains, and Preparation of Triton X-Extracted Antigen

Each experimental or control group comprised of five 6–8-week-old female mice of the strains BALB/c, C57BL/6, B cell-deficient (Igh-6^tm1Cgn^), C3H/HeJ, and C3H/HePAS. The animals were housed under pathogen-free conditions in the Animal Research Facilities of the Medical School of Ribeirão Preto, USP. Three independent experiments were carried out to generate a result, except for the construction of the cumulative survival curve, which was performed once.

The *vapG* antigen sequence was synthesized by PCR-amplification of a 519-bp DNA fragment (comprising the *vapG* sequence) from the *R. equi* virulence plasmid (ATCC 33701). Primers (*vapG*-Fw, 5′-GCGGCCGTCGACAAGAGAGGATGATATCATGAGT-3′; *vapG*-Rv, 5′-GCGCGCTGCAGCTATTGCCACCCTCCGGTTC-3′) were used to generate *Sal*I and *Bam*HI restriction sites at either end of the DNA fragment, so as to facilitate directed insertion of *vapG* into the pYA3137 plasmid, as reported by Oliveira et al. (2007).

Both the attenuated *S. enterica* Typhimurium 3987 strains [carrying either *vapG*+, *vapA*+, or the empty vector (control *vapA*-)] and the virulent strain of *R. equi* (ATCC 33701) were grown and prepared as described by Oliveira et al. (2010). Triton X-extracted antigen (APTX) was prepared as described previously by Tan et al. (1995).

### Immunization and Challenge Protocols

Mice were orally immunized with attenuated *Salmonella* harboring VapG+ on days 0 and 14 of the experiment as described previously by Oliveira et al. (2007). PBS and *Salmonella* carrying empty vector were orally administrated to the negative control mice. Challenges with *R. equi* were conducted by administrating inoculum of the virulent *R. equi* strain ATCC 33701 at a sub-lethal dose, 30 days after the first immunization. Organs were harvested 5 days after the challenge with *R. equi*.

The curve of the cumulative mice survival was constructed by using the Kaplan-Meier method (Kaplan and Meier, 1958). Thirty days after immunization with a single oral vaccine, mice were challenged with a lethal *R. equi* inoculum. Mortality was recorded daily during the 15-day period after the challenge.

### Quantification of Bacterial Burden in Organs of *R. equi*-Challenged Mice

Quantification of viable *R. equi* recovered from the spleen and liver of the challenged mice was performed as previously described (Oliveira et al., 2007). Briefly, 30 days after the first immunization, mice were infected intravenously with 4 × 10^6^ colony forming units (CFUs) of virulent *R. equi*. Five days after the challenge, the spleen and liver from the mice were harvested and aseptically homogenized. Samples (100 μL each) of the homogenates were diluted in sterile PBS, plated onto BHI agar in duplicates, and incubated at 37°C for 36 h before CFU counting.

### Cytokine Determination

Samples of the spleen homogenates obtained 30 days after the first immunization or 5 days after *R. equi* challenge were assessed for IL- 12p70, IFN-γ, and TNF-α levels by ELISA, using an OptEIA kit (BD Pharmingen, San Diego, CA, USA) according to the manufacturer’s instructions.

### Flow Cytometry Analysis

The spleen cells (1 × 10^7^) from immunized and control mice were harvested 15 days post-immunization, washed with ice-cold PBS, and incubated (30 min, 4°C) with anti-CD16/CD32 mAb (Fc block, clone 2.4G2, BD Pharmingen). After centrifugation and washing, the cells were incubated with anti-CD19, anti-CD3, anti-CD4, and anti-CD8 (PE- or FITC-labeled; BD Pharmingen) for 40 min. Washing was performed using PBS with 0.5% BSA, and the cells were analyzed using a Guava flow cytometer and CytoSoft version 4.2.1 software (Millipore, Billerica, MA, USA).

### Detection of Anti-*R. equi* Specific Antibodies in Mice Serum

Blood samples were collected from mice (*n* = 4) on days 0 and 30 after the first immunization. Serum anti-*R. equi* antibodies were titrated by ELISA in APTX- (primarily constituted of VapA as demonstrated by Prescott et al., 1997b) or recombinant VapG-coated microtiter plates (Costar, USA) prepared as described previously by Okoko et al. (2015) (Supplementary Figure S1). Samples were diluted 1:120 in PBS containing 0.05% Tween-20 for the detection of anti-APTX IgG, and serially (log_2_) for the titration of anti-VapG IgG. Reactions were detected using goat anti-mouse IgG conjugated with horseradish-peroxidase (1:5000, Santa Cruz Biotechnology).

### Statistical Analyses

Statistical analysis was performed using GraphPad Prism 6 software. Comparison of data between the two groups was performed by Student’s *t*-test. Data from three or more groups were compared by one-way analysis of variance (ANOVA) followed by Tukey’s test. The index of mice survival for each group was analyzed by Log-rank test (Peto et al., 1977).

## Results

### Immunization with VapG+ Carried by Attenuated *Salmonella* Protects Mice against *R. equi* Infection

**FIGURE 1:**
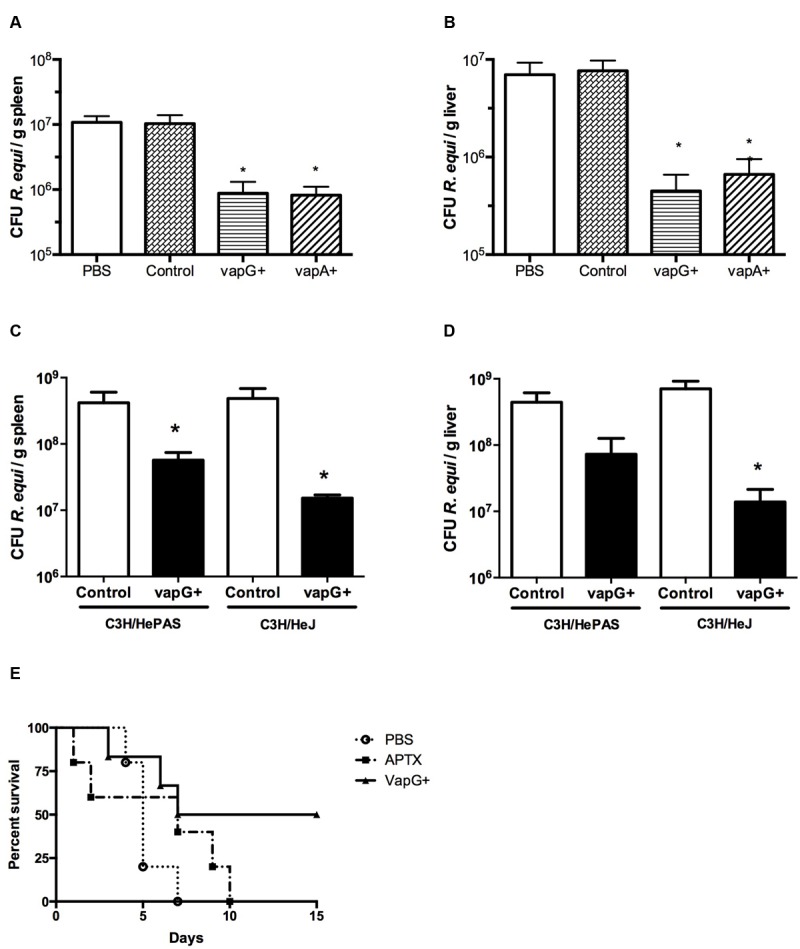
**Vaccination with attenuated *Salmonella enterica* Typhimurium carrying VapG antigen induces protection against *Rhodococcus equi* challenge in mice.** Mice were orally immunized on days 0 and 14 with either *S. enterica* Typhimurium χ3987-pYA3137 (Control), χ3987-pYA3137*vapG* (VapG+), χ3987-pYA3137*vapA* (VapA+), PBS, or APTX (with Freund’s adjuvant). Thirty days after the first immunization, mice were challenged with *R. equi* (ATCC33701 strain), before being sacrificed 5 days post-infection. The *R. equi* burden was evaluated in the spleen and liver of **(A,B)** BALB/c, and **(C,D)** C3H/HePAS and C3H/HeJ mice. **(E)** A cumulative survival curve was constructed for *R. equi*-challenged BALB/c mice following immunization with PBS (circles), VapG+ (triangles), or APTX (squares). Data are represented as mean ± SE, ^∗^*p* < 0.05 (Tukey’s test).

It was previously demonstrated that oral immunization of mice with *Salmonella*-*vapA*+ confers resistance toward *R. equi* infection (Oliveira et al., 2007, 2010; Cardoso et al., 2013). Because VapG antigen, similar to VapA, is encoded by a gene of the *R. equi* virulence plasmid and is highly expressed in the lung tissue of *R. equi*-infected foals (Coulson et al., 2010), in this study, we evaluated the effect of *Salmonella*-*vapG*+ vaccination in mice. A significant reduction in the bacterial burden in the spleen and liver, the *R. equi*-targeted organs, was detected in the vaccinated mice challenged with a *R. equi* inoculum at a sub-lethal dose as compared to that detected in the unvaccinated mice (**Figures 1A,B**). Interestingly, the observed CFU reduction in *Salmonella*-*vapG*+-immunized mice was comparable to that previously observed in *Salmonella*-*vapA*+-immunized mice (Oliveira et al., 2007). Furthermore, in this study, the protective effect of the Toll-like Receptor 4 (TLR4) activation due to the *Salmonella* carrier occurred in an independent manner, since the results in the TLR4-deficient mice were similar to those in the WT mice (**Figures 1C,D**). When challenged with a lethal dose of *R. equi*, 50% of the *Salmonella*-*vapG*+-immunized mice survived, whereas all non-immunized control mice, who received PBS or the empty vector instead of the vaccine, died within 1 week after the challenge. Mice immunized with APTX, which induces a strong anti-VapA humoral response (Prescott et al., 1997b), died within 10 days after the challenge (**Figure 1E**).

### *Salmonella*-*vapG*+ Vaccination Elicits Interferon-Gamma Responses in Mice

**FIGURE 2:**
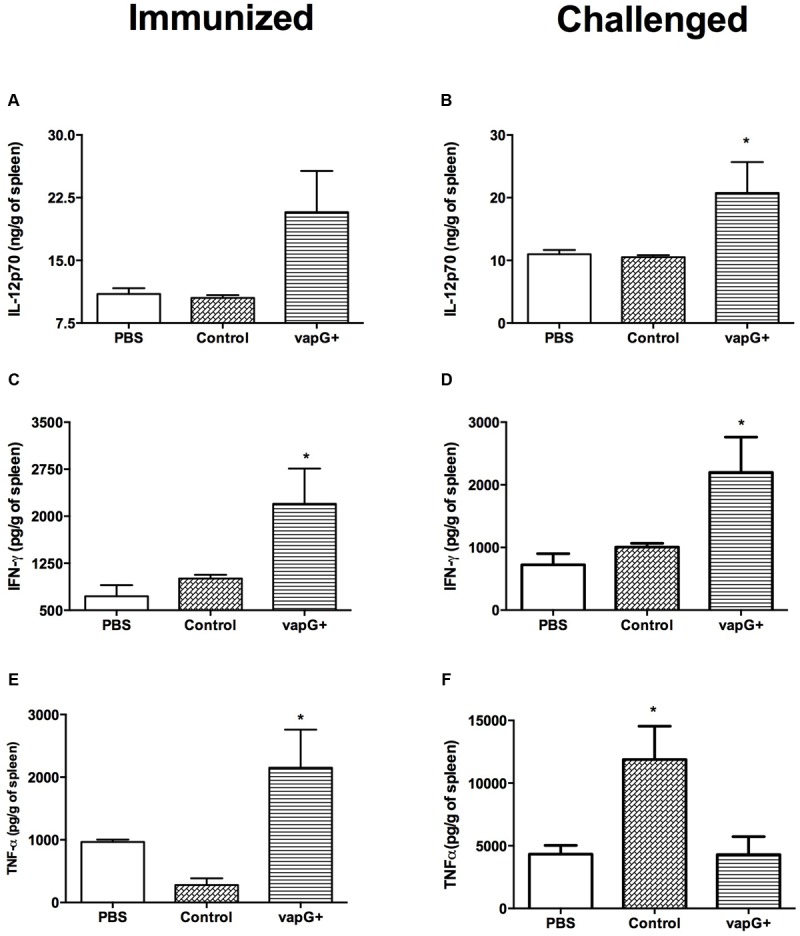
**Vaccination with attenuated *S. enterica* Typhimurium carrying VapG antigen elicits a Th1 immune response.** Mice were orally immunized on days 0 and 14 with *S. enterica* Typhimurium χ3987-pYA3137 (Control), χ3987-pYA3137*vapG* (VapG+), or PBS. Thirty days after the first immunization, mice were challenged with *R. equi* (ATCC33701 strain), before being sacrificed 5 days post-infection. The cytokines **(A,B)** IL12p70, **(C,D)** IFN-γ, and **(E,F)** TNF-α were evaluated by ELISA using the spleen homogenates. Data are represented as mean ± SE, *n* = 5 mice/treatment group, ^∗^*p* < 0.05 (Tukey’s test).

To evaluate the association between the protection conferred by *Salmonella*-*vapG*+ vaccination and a cytokine response, the content of Th1-type cytokines (i.e., IL-12, IFN-γ, and TNF-α) in the spleen of vaccinated and *R. equi*-challenged mice was assessed. The spleen homogenates from *Salmonella*-*vapG*+-immunized mice exhibited a higher IL-12 and IFN-γ content than that in the spleen homogenates from mice in the control group, before or after the *R. equi* challenge (**Figures 2A–D**). The TNF-α content in the spleen was also augmented following *Salmonella*-*vapG*+ vaccination (**Figure 2E**). However, the TNF-α content decreased when the immunized mice were challenged with *R. equi*, reaching to levels comparable to those in the mice from the negative control group (**Figure 2F**). Notably, the control group vaccinated with the empty vector (*Salmonella-vapA*-) augmented the TNF-α content in the spleen significantly, and this result was consistent with that of the previous studies (Oliveira et al., 2010; Cardoso et al., 2013).

### *Salmonella*-*vapG*+ Vaccination Increases B-Cell Populations in the Spleen and Induces a Protective Humoral Response in Mice

**FIGURE 3:**
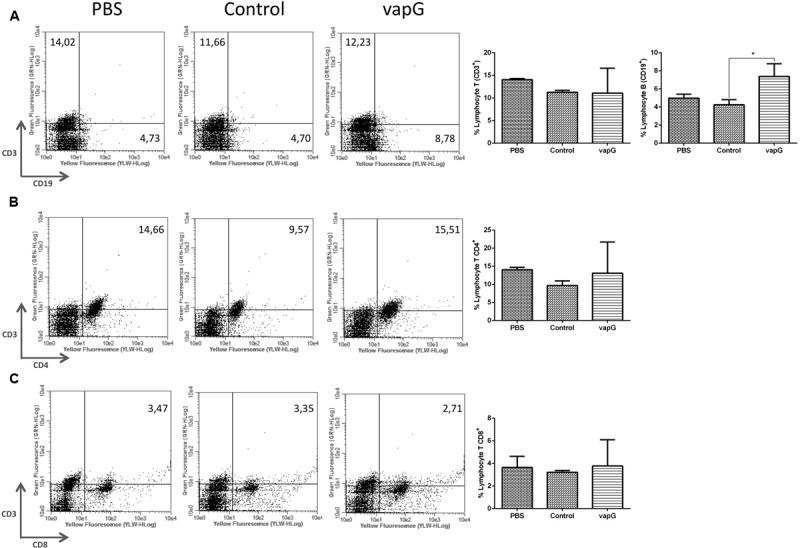
**Vaccination with attenuated *S. enterica* Typhimurium carrying VapG antigen increases B cell frequency.** BALB/c mice were orally immunized on days 0 and 14 with *S. enterica* Typhimurium χ3987-pYA3137 (Control), χ3987-pYA3137*vapG* (VapG+), or PBS. Thirty days after the first immunization, mice were sacrificed, and the frequency of splenic **(A)** CD3+ and CD19+, **(B)** CD3+CD4+, and **(C)** CD3+CD8+ cells was evaluated. Data represent the mean ± SE, *n* = 5 mice/treatment group, ^∗^*p* < 0.05 (Tukey’s test).

To determine the effect of *Salmonella*-*vapG*+ vaccination on the lymphocyte proliferation, the frequency of CD19+, CD3+CD4+, and CD3+CD8+ cells was compared in spleen homogenates from immunized and control group mice. The flow cytometric analysis showed that as compared to mice in the PBS- or *Salmonella*-*vapA*–control groups, *Salmonella*-*vapG*+-immunized mice displayed an increased frequency of B cells (**Figure 3A**), whereas the incidence of T cells was similar among the groups (**Figures 3B,C**).

**FIGURE 4:**
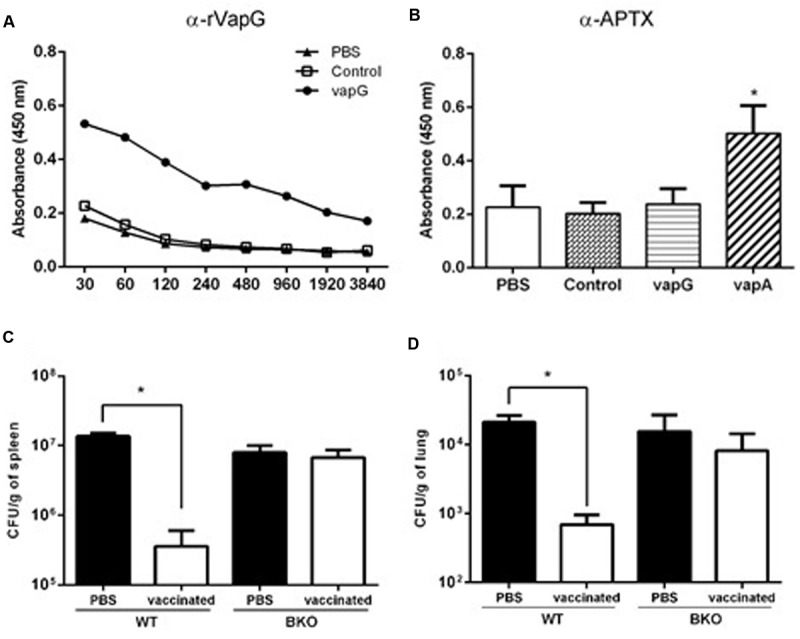
**Vaccination with attenuated *S. enterica* Typhimurium carrying VapG antigen induces antibodies critical to the protective immune response.** BALB/c mice were orally immunized on days 0 and 14 with *S. enterica* Typhimurium χ3987-pYA3137 (Control), χ3987-pYA3137*vapA* (VapA+), χ3987-pYA3137*vapG* (VapG+), or PBS. Serum was collected 30 days after the first immunization, and used to measure **(A)** VapG- or **(B)** APTX-specific IgG by ELISA. C57BL/6 BKO and C57BL/6 WT mice were orally immunized on days 0 and 14 with *S. enterica* Typhimurium χ3987-pYA3137*vapG* (VapG+) or PBS. Thirty days after the first immunization, mice were challenged with *R. equi* (ATCC 33701 strain), before being sacrificed 5 days post-infection. The *R. equi* burden was evaluated in the **(C)** spleen (CFU/g) and **(D)** lung. Data represent the mean ± SE, ^∗^*p* < 0.05 (Tukey’s test).

To evaluate the specific antibodies secreted due to *Salmonella*-*vapG*+ vaccination, serum titration was performed using recombinant VapG-coated microplates. As shown in **Figure 4A**, the serum IgG of the *Salmonella*-*vapG*+-vaccinated mice reacted with the recombinant VapG, showing absorbance readings four-times higher than those obtained by reacting with serum IgG from negative controls, up to a dilution of 1:960.

It was previously reported that Vap proteins display high homology in their C-terminus regions (Takai et al., 2000); therefore, the cross-reactivity of serum IgG from the *Salmonella*-*vapG*+-vaccinated mice with recombinant VapA antigen, contained in the APTX preparation used to coat a second set of microplates, was assessed. For this analysis, the serum samples were used at a dilution of 1:120, and serum from *Salmonella*-*vapA*+-immunized mice served as the positive control. Only this positive control serum reacted with the APTX coating (**Figure 4B**), which was not recognized by the serum IgG of the *Salmonella*-*vapG*+-vaccinated mice. This suggests that the vaccine induced generation of VapG-specific IgG antibodies, which account for the protective humoral response against *R. equi* infection as studied in the vaccinated mice.

To address the relevance of the elicited antibody response for protection against *R. equi* infection conferred by *Salmonella*-*vapG*+ vaccination, B-cell deficient mice (B-cell KO) were immunized (Kitamura et al., 1991). PBS, instead *Salmonella*-*vapG*+, was administered in the negative control mice. The *Salmonella*-*vapG*+-vaccinated B-cell KO mice, as well as the negative control mice, were not protected against the *R. equi* challenge, as demonstrated by a similar bacterial burden displayed by all the groups of mice (**Figures 4C,D**). Thus, the generation of VapG-specific antibodies is crucial for the protective effect against *R. equi* conferred by *Salmonella*-*vapG*+ vaccination in mice.

## Discussion

For several years, vaccines containing the bacterial virulence antigen VapA (encoded by an 85-kb plasmid) were considered as the most promising strategy to prevent rhodococcosis. However, this strategy has been unsuccessful until now owing to the complexity of *R. equi* virulence, as observed in previous studies performed using different strategies in immunized horses—virulence plasmid-cured *R. equi* strain (Giguere et al., 1999), inactivated *R. equi* strain (van der Geize et al., 2011; Bordin et al., 2014), or VapA associated to other antigens (Cauchard et al., 2014). Some strategies, such as using a DNA vaccine (Phumoonna et al., 2008) or virulence plasmid-negative *R. equi* strain expressing *vapA* (Whitehead et al., 2012), were unsuccessful even in mouse models. Coulson et al. (2010), by using a *vapG*-mutant strain, reported that VapG plays a major role in *R. equi* virulence. Nevertheless, the efficacy of VapG as an *R. equi* vaccine candidate has not yet been tested.

In the present study, we assessed VapG as a *Salmonella*-based live-vector vaccine, an approach that was previously efficient in delivering VapA (Oliveira et al., 2007, 2010; Cardoso et al., 2013). Vaccination with *Salmonella*-*vapG*+ significantly reduced the bacterial burden exhibited by *R. equi*-challenged mice. However, among the mice challenged with a lethal dose of *R. equi* inoculum, only 50% of the *Salmonella*-*vapG*+-vaccinated mice survived, while 100% survival rate was reported for *Salmonella*-*vapA*+-vaccinated mice (Oliveira et al., 2007). Vaccination with either *Salmonella*-*vapG*+ or *Salmonella*-*vapA*+ was associated with a significant increase in IL-12 and IFN-γ production in response to *R. equi* infection, whereas the TNF-α levels produced in *Salmonella*-*vapA*+- or *vapG*+-vaccinated mice were as low as those in the negative control mice. Immunization with *Salmonella*-*vapG*+ caused an increase in the splenic B-cell population, whereas the frequency of CD4+ and CD8+ T cells did not vary amongst the groups. This result contrasts previously reported findings for *Salmonella*-*vapA*+-vaccinated mice (Oliveira et al., 2010) that exhibited a significant increase in T-cell populations. Our observations suggested that the *Salmonella*-*vapG*+ vaccine elicits a protective humoral immune response, but not a cellular immune response, a fact that may explain the modest survival rate among the *Salmonella*-*vapG*+-vaccinated mice that were challenged with a lethal dose of *R. equi* inoculum. The *Salmonella*-*vapG*+-vaccinated mice were shown to produce specific antibodies that account for a partial protection against *R. equi* infection, a hypothesis that is supported by the failure of the *Salmonella*-*vapG*+ vaccine in protecting B-cell KO mice against *R. equi* infection.

It is accepted that VapA+ is a fundamental component of any effective *R. equi* vaccine because it elicits a robust Th1 cellular immune response, which is essential for fighting against the bacteria. Simultaneously, the humoral immune response elicited by VapA seems inefficient to protect the challenged mice (Prescott et al., 1997b). Our present study demonstrates that VapG+ elicits a specific humoral immune response, and makes the relevance of this response evident in conferring partial protection against *R. equi* infection. Then, we postulate that a vaccine against *R. equi* would be more efficient by associating various Vap antigens. The combined delivery of VapA and VapG antigens may result in protective responses owing to the stimulation of both axes of immunity—the cellular axis by VapA and the humoral axis by VapG. Presumably, the double efficient stimuli would provide an advantageous vaccination strategy, increasing the chances of reproducibility in foals similar to that obtained in mouse models. Nonetheless, we must highlight that several vaccination strategies that were successful in laboratory experimental animals failed in animals of the target (equine) species, as previously demonstrated with unsuccessful immunization of foals with VapA (Prescott et al., 1997a; Lohmann et al., 2013), a formulation that had proved protective in mice (Prescott et al., 1997b; Haghighi and Prescott, 2005). Because prevention and treatment of rhodococcosis in foals is frequently carried out by passive immunization, achieved by the administration of hyperimmune plasma (HIP) or vaccination of pregnant mares, we proposed to include VapG, rather than VapA, in the immunogenic preparations used to induce formation of specific antibodies to be transferred to foals. Various commercial HIPs contain several different subclasses of immunoglobulins that are specific to VapA+ or components of inactivated virulent *R. equi*. Although the administration of the commercial HIPs has been reported to be associated mild pneumonia following *R. equi* challenge (Sanz et al., 2016), their efficacy is still controversy (Giguere et al., 2002; Caston et al., 2006; Sanz et al., 2014). Commercial HIPs elicit a variable range of responses, probably due to the presence of several Ig isotypes with specificities toward numerous antigens used in the immunization procedure. The use of VapG as an immunogen may provide an approach to improve the efficacy of the treatment with HIP of *R. equi*. Briefly, we are trying to offer a rational basis for VapG addition in future vaccines against rhodococcosis and to make passive immunization of foals more efficient.

## Conclusion

The results of the present study allow us to propose VapG as an appropriate antigen for the development of vaccines and treatment against *R. equi* infection. This hypothesis is based on the ability of VapG to elicit a robust humoral immune response that can confer partial protection to *R. equi*-challenged mice. Further investigation is required to select the ideal delivery of VapA and VapG antigens in a single vaccine, and study the cellular and humoral responses elicited by the vaccine in newborn foals exposed to *R. equi* infection.

## Author Contributions

Conceived and designed the experiments: MT, EH, AO, SC, MR-B, SS. Performed the experiments: MT, AO, SC. Analyzed the data: MT, EH, AO, SC, SS. Wrote the paper: MT, MR-B, SS. All authors have read and approved the final manuscript.

## Conflict of Interest Statement

The authors declare that the research was conducted in the absence of any commercial or financial relationships that could be construed as a potential conflict of interest.

## References

[B1] Benoit S. Benachour A. Taouji S. Auffray Y. Hartke A. (2002). H_2_O_2_, which causes macrophage-related stress, triggers induction of expression of virulence-associated plasmid determinants in *Rhodococcus equi*. *Infect. Immun*. 70 3768–3776. 10.1128/IAI.70.7.3768-3776.2002 12065520 PMC128077

[B2] Bordin A. I. Pillai S. D. Brake C. Bagley K. B. Bourquin J. R. Coleman M. (2014). Immunogenicity of an electron beam inactivated *Rhodococcus equi* vaccine in neonatal foals. *PLoS ONE* 9:e105367 10.1371/journal.pone.0105367 PMC4143214 25153708

[B3] Cardoso S. A. Oliveira A. F. Ruas L. P. Trevisani M. M. De Oliveira L. L. Hanna E. S. (2013). Nasal vaccination with attenuated *Salmonella* expressing VapA: TLR2 activation is not essential for protection against *R. equi* infection. *Vaccine* 31 4528–4535. 10.1016/j.vaccine.2013.07.067 23933366

[B4] Caston S. S. McClure S. R. Martens R. J. Chaffin M. K. Miles K. G. Griffith R. W. (2006). Effect of hyperimmune plasma on the severity of pneumonia caused by *Rhodococcus equi* in experimentally infected foals. *Vet. Ther*. 7 361–375. 17216591

[B5] Cauchard S. Bertrand F. Barrier-Battut I. Jacquet S. Laurentie M. Barbey C. (2014). Assessment of the safety and immunogenicity of *Rhodococcus equi*-secreted proteins combined with either a liquid nanoparticle (IMS 3012) or a polymeric (PET GEL A) water-based adjuvant in adult horses and foals–identification of promising new candidate antigens. *Vet. Immunol. Immunopathol*. 157 164–174. 10.1016/j.vetimm.2013.12.003 24445196

[B6] Coulson G. B. Agarwal S. Hondalus M. K. (2010). Characterization of the role of the pathogenicity island and vapG in the virulence of the intracellular actinomycete pathogen *Rhodococcus equi*. *Infect. Immun*. 78 3323–3334. 10.1128/IAI.00081-10 20439471 PMC2916281

[B7] Giguere S. Gaskin J. M. Miller C. Bowman J. L. (2002). Evaluation of a commercially available hyperimmune plasma product for prevention of naturally acquired pneumonia caused by *Rhodococcus equi* in foals. *J. Am. Vet. Med. Assoc*. 220 59–63. 10.2460/javma.2002.220.59 12680449

[B8] Giguere S. Hondalus M. K. Yager J. A. Darrah P. Mosser D. M. Prescott J. F. (1999). Role of the 85-kilobase plasmid and plasmid-encoded virulence-associated protein A in intracellular survival and virulence of *Rhodococcus equi*. *Infect. Immun*. 67 3548–3557. 10377138 10.1128/iai.67.7.3548-3557.1999 PMC116543

[B9] Giguere S. Jacks S. Roberts G. D. Hernandez J. Long M. T. Ellis C. (2004). Retrospective comparison of azithromycin, clarithromycin, and erythromycin for the treatment of foals with *Rhodococcus equi* pneumonia. *J. Vet. Intern. Med*. 18 568–573. 10.1111/j.1939-1676.2004.tb02587.x 15320600

[B10] Giguere S. Prescott J. F. (1997). Clinical manifestations, diagnosis, treatment, and prevention of *Rhodococcus equi* infections in foals. *Vet. Microbiol*. 56 313–334. 10.1016/S0378-1135(97)00099-0 9226845

[B11] Giles C. Vanniasinkam T. Ndi S. Barton M. D. (2015). *Rhodococcus equi* (*Prescottella equi*) vaccines; the future of vaccine development. *Equine Vet. J*. 47 510–518. 10.1111/evj.12310 24945608

[B12] Haghighi H. R. Prescott J. F. (2005). Assessment in mice of vapA-DNA vaccination against *Rhodococcus equi* infection. *Vet. Immunol. Immunopathol*. 104 215–225. 10.1016/j.vetimm.2004.12.006 15734542

[B13] Jacks S. Giguere S. Prescott J. F. (2007). In vivo expression of and cell-mediated immune responses to the plasmid-encoded virulence-associated proteins of *Rhodococcus equi* in foals. *Clin. Vaccine Immunol*. 14 369–374. 10.1128/CVI.00448-06 17301216 PMC1865619

[B14] Jain S. Bloom B. R. Hondalus M. K. (2003). Deletion of vapA encoding virulence associated protein A attenuates the intracellular actinomycete *Rhodococcus equi*. *Mol. Microbiol*. 50 115–128. 10.1046/j.1365-2958.2003.03689.x 14507368

[B15] Kaplan E. L. Meier P. (1958). Nonparametric-estimation from incomplete observations. *J. Am. Stat. Assoc*. 53 457–481. 10.2307/2281868

[B16] Kitamura D. Roes J. Kuhn R. Rajewsky K. (1991). A B cell-deficient mouse by targeted disruption of the membrane exon of the immunoglobulin μ chain gene. *Nature* 350 423–426. 10.1038/350423a0 1901381

[B17] Lohmann K. L. Lopez A. M. Manning S. T. Marques F. J. Brownlie R. Allen A. L. (2013). Failure of a VapA/CpG oligodeoxynucleotide vaccine to protect foals against experimental *Rhodococcus equi* pneumonia despite induction of VapA-specific antibody and interferon-gamma response. *Can. J. Vet. Res.* 77 161–169. 24101791 PMC3700440

[B18] Monego F. Maboni F. Krewer C. Vargas A. Costa M. Loreto E. (2009). Molecular characterization of *Rhodococcus equi* from horse-breeding farms by means of multiplex PCR for the vap gene family. *Curr. Microbiol.* 58 399–403. 10.1007/s00284-009-9370-6 19205798

[B19] Okoko T. Blagova E. V. Whittingham J. L. Dover L. G. Wilkinson A. J. (2015). Structural characterisation of the virulence-associated protein VapG from the horse pathogen *Rhodococcus equi*. *Vet. Microbiol.* 179 42–52. 10.1016/j.vetmic.2015.01.027 25746683 PMC4518536

[B20] Oliveira A. F. Ferraz L. C. Brocchi M. Roque-Barreira M. C. (2007). Oral administration of a live attenuated *Salmonella* vaccine strain expressing the VapA protein induces protection against infection by *Rhodococcus equi*. *Microbes Infect.* 9 382–390. 10.1016/j.micinf.2006.12.019 17307012

[B21] Oliveira A. F. Ruas L. P. Cardoso S. A. Soares S. G. Roque-Barreira M. C. (2010). Vaccination of mice with *salmonella* expressing VapA: mucosal and systemic Th1 responses provide protection against *Rhodococcus equi* infection. *PLoS ONE* 5:e8644 10.1371/journal.pone.0008644 PMC2800180 20072623

[B22] Peto R. Pike M. C. Armitage P. Breslow N. E. Cox D. R. Howard S. V. (1977). Design and analysis of randomized clinical trials requiring prolonged observation of each patient. II. analysis and examples. *Br. J. Cancer* 35 1–39. 10.1038/bjc.1977.1 831755 PMC2025310

[B23] Phumoonna T. Barton M. D. Vanniasinkam T. Heuzenroeder M. W. (2008). Chimeric vapA/groEL2 DNA vaccines enhance clearance of *Rhodococcus equi* in aerosol challenged C3H/He mice. *Vaccine* 26 2457–2465. 10.1016/j.vaccine.2008.03.015 18423949

[B24] Prescott J. F. Nicholson V. M. Patterson M. C. Zandona Meleiro M. C. Caterino de Araujo A. Yager J. A. (1997a). Use of *Rhodococcus equi* virulence-associated protein for immunization of foals against *R. equi* pneumonia. *Am. J. Vet. Res.* 58 356–359. 9099378

[B25] Prescott J. F. Patterson M. C. Nicholson V. M. Morein B. Yager J. A. (1997b). Assessment of the immunogenic potential of *Rhodococcus equi* virulence associated protein (VapA) in mice. *Vet. Microbiol.* 56 213–225. 9226836 10.1016/s0378-1135(97)00090-4

[B26] Ren J. Prescott J. F. (2003). Analysis of virulence plasmid gene expression of intra-macrophage and in vitro grown *Rhodococcus equi* ATCC 33701. *Vet. Microbiol.* 94 167–182. 10.1016/S0378-1135(03)00099-3 12781484

[B27] Sanz M. G. Loynachan A. Horohov D. W. (2016). *Rhodococcus equi* hyperimmune plasma decreases pneumonia severity after a randomised experimental challenge of neonatal foals. *Vet. Rec.* 178:261 10.1136/vr.103095 26932206

[B28] Sanz M. G. Oliveira A. F. Page A. Horohov D. W. (2014). Administration of commercial *Rhodococcus equi* specific hyperimmune plasma results in variable amounts of IgG against pathogenic bacteria in foals. *Vet. Rec.* 175:485 10.1136/vr.102594 25117301

[B29] Takai S. Hines S. A. Sekizaki T. Nicholson V. M. Alperin D. A. Osaki M. (2000). DNA sequence and comparison of virulence plasmids from *Rhodococcus equi* ATCC 33701 and 103. *Infect. Immun.* 68 6840–6847. 10.1128/IAI.68.12.6840-6847.2000 11083803 PMC97788

[B30] Tan C. Prescott J. F. Patterson M. C. Nicholson V. M. (1995). Molecular characterization of a lipid-modified virulence-associated protein of *Rhodococcus equi* and its potential in protective immunity. *Can. J. Vet. Res.* 59 51–59. 7704843 PMC1263734

[B31] van der Geize R. Grommen A. W. Hessels G. I. Jacobs A. A. Dijkhuizen L. (2011). The steroid catabolic pathway of the intracellular pathogen *Rhodococcus equi* is important for pathogenesis and a target for vaccine development. *PLoS Pathog.* 7:e1002181 10.1371/journal.ppat.1002181 PMC3161971 21901092

[B32] Venner M. Rodiger A. Laemmer M. Giguere S. (2012). Failure of antimicrobial therapy to accelerate spontaneous healing of subclinical pulmonary abscesses on a farm with endemic infections caused by *Rhodococcus equi*. *Vet. J.* 192 293–298. 10.1016/j.tvjl.2011.07.004 21924651

[B33] Whitehead A. E. Parreira V. R. Hewson J. Watson J. L. Prescott J. F. (2012). Development of a live, attenuated, potential vaccine strain of *R. equi* expressing vapA and the virR operon, and virulence assessment in the mouse. *Vet. Immunol. Immunopathol.* 145 479–484. 10.1016/j.vetimm.2011.10.011 22088674

[B34] Yamshchikov A. V. Schuetz A. Lyon G. M. (2010). *Rhodococcus equi* infection. *Lancet Infect. Dis.* 10 350–359. 10.1016/S1473-3099(10)70068-2 20417417

[B35] Zink M. C. Yager J. A. Prescott J. F. Fernando M. A. (1987). Electron microscopic investigation of intracellular events after ingestion of *Rhodococcus equi* by foal alveolar macrophages. *Vet. Microbiol.* 14 295–305. 10.1016/0378-1135(87)90117-9 3672872

