# Supplementary material for: Vaccination of Mice with Virulence-Associated Protein G (VapG) Antigen Confers Partial Protection against Rhodococcus equi Infection through Induced Humoral Immunity
Source: Front Microbiol. 2017 May 11;8:857. doi: 10.3389/fmicb.2017.00857 (PMC5425581; doi:10.3389/fmicb.2017.00857)
Supplement: Supplementary file 1 [file Image_1.PDF]

Appendix A: Supplementary material

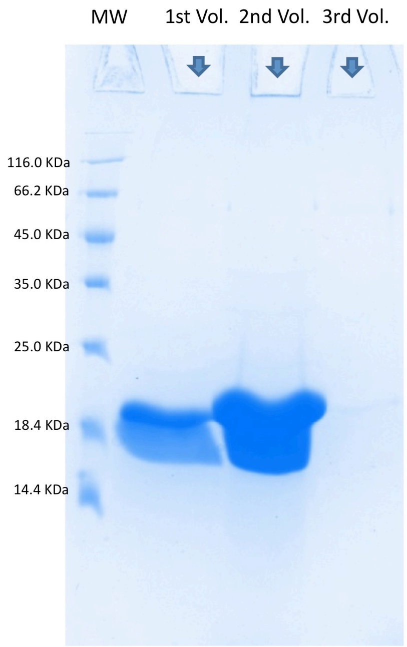

Fig S1: VapG sequence was synthesized from Epoch Life Science (Texas, USA) and cloned into NdeI/XhoI digested pET28a. The plasmid was used to transform BL21-Gold(DE3) competent cells (Agilent Technologies, California, USA) and recombinant protein was produced using the protocol of fermentation described by Okoko et al (2015). Cells from 500 mL of culture were pelleted by centrifugation and lysed by sonication (6 pulses of 20 seconds). After new centrifugation in high speed, supernatant was supplemented with 50mM of Imidazole and applied in Histrap column (GE Life Sciences, UK) and eluted with PBS added 300 mM imidazole. Recombinant VapG was eluted in two volumes of column as showed in the figure.
